# Effects of the Population-Based “10,000 Steps Duesseldorf” Intervention for Promoting Physical Activity in Community-Dwelling Adults: Protocol for a Nonrandomized Controlled Trial

**DOI:** 10.2196/39175

**Published:** 2022-09-21

**Authors:** Paula M Matos Fialho, Liane Günther, Elena Schmitz, Jannis Trümmler, Sorina Willemsen, Markus Vomhof, Andrea Icks, Alexander Lang, Oliver Kuss, Simone Weyers, Claudia R Pischke

**Affiliations:** 1 Institute of Medical Sociology Centre for Health and Society, Medical Faculty Heinrich Heine University Duesseldorf Duesseldorf Germany; 2 Institute for Health Services Research and Health Economics, Centre for Health and Society, Medical Faculty Heinrich Heine University Duesseldorf Duesseldorf Germany; 3 Institute for Health Services Research and Health Economics, German Diabetes Center, Leibniz Center for Diabetes Research Heinrich Heine University Duesseldorf Duesseldorf Germany; 4 German Center for Diabetes Research, Partner Düsseldorf München-Neuherberg Germany; 5 Institute for Biometrics and Epidemiology, German Diabetes Center, Leibniz Institute for Diabetes Research Heinrich Heine University Duesseldorf Duesseldorf Germany; 6 Centre for Health and Society, Medical Faculty Heinrich Heine University Duesseldorf Duesseldorf Germany

**Keywords:** physical activity, population-based complex intervention, replication study, multilevel strategy

## Abstract

**Background:**

The World Health Organization recommends 150 minutes of moderate to vigorous physical activity (PA), which translates to approximately 7000 to 10,000 steps per day for adults. In Germany, less than half of the population in this age range meets this recommendation, highlighting the need for population-based intervention approaches for promoting daily PA.

**Objective:**

The complex community-based PA intervention “10,000 Steps Ghent,” which was originally developed in Belgium and was shown to be effective for PA promotion, has been adapted for implementation and evaluation in 2 German cities. The original Belgian study is currently being replicated, and we aim to examine the effectiveness of the adapted intervention among adults living in intervention city districts in Duesseldorf when compared with those living in control city districts in Wuppertal, over the course of 1 year.

**Methods:**

A controlled intervention trial examining the effects of an intervention addressing multiple levels (eg, individual level: website; organizational level: PA promotion in companies; community level: media campaigns and environmental changes) is being conducted. PA and various secondary outcomes will be assessed in 2 random samples of adults aged 25 to 75 years (n=399 in each city) at baseline and after 1 year.

**Results:**

Funding for this study was obtained in March 2020. Recruitment for this study and baseline data collection were conducted from May 2021 to March 2022 (as of March 2022, 626 participants were enrolled in the study). The intervention will be implemented in Duesseldorf for 1 year from April 2022 onward, and follow-up assessments will be conducted, starting in May 2023 (until September 2023). Data analysis will be performed in fall 2023, and the results will be published in spring 2024.

**Conclusions:**

To our knowledge, this is the first research project (currently underway in Germany) that is aimed at replicating the effects of a complex intervention for PA promotion that was previously shown to be effective in another European country.

**Trial Registration:**

German Clinical Trials Register DRKS00024873; https://tinyurl.com/4c9e8azh

**International Registered Report Identifier (IRRID):**

DERR1-10.2196/39175

## Introduction

Physical inactivity is the fourth largest risk factor for mortality worldwide and is one of the main drivers contributing to the etiology of noncommunicable diseases, such as type 2 diabetes, cardiovascular diseases, and certain cancers, which are preventable to a certain extent [1]. In Germany, an estimated 91% of deaths are due to these noncommunicable diseases [2]. It is widely known from previous research that the reduction of an inactive lifestyle [3] and an increase in regular physical activity (PA) leads to improvements in health, including physical, psychological, cognitive, and functional health over the entire life span [3,4]. A recently published cohort study found that among middle-aged men and women walking ≥7000 steps per day was associated with lower mortality rates compared with those walking ≤7000 steps per day [5].

The 2020 update of the recommendations for PA of the World Health Organization (WHO) and the American College of Sports Medicine states that “all adults should undertake 150-300 minutes of moderate-intensity, or 75-150 minutes of vigorous-intensity physical activity, or some equivalent combination of moderate-intensity and vigorous-intensity aerobic physical activity, per week” [6]. It has been noted that 150 minutes per week is the equivalent of 7000 to 10,000 steps walked per day [7,8]. In addition, muscular strengthening PA on at least two days per week is recommended to achieve the health benefits mentioned earlier and reduce the risks of chronic diseases (and the associated cost-intensive curative and rehabilitative measures) [6]. Furthermore, Rütten and Pfeifer [9] emphasize in the German national PA recommendations that adults should also avoid long, uninterrupted sitting phases and, if possible, interrupt sitting regularly with PA. According to the authors, “the greatest health benefits take place when individuals who were entirely physically inactive become somewhat more active. This means that all additional PA is linked to health benefits. Every single step away from physical inactivity is important, no matter how small, and promotes health” [9]. Currently, in Germany, only 43% of women and 48% of men aged ≥18 years meet the WHO and American College of Sports Medicine recommendations for PA [10], highlighting the need for population-based intervention approaches to promote daily PA.

The results of a meta-analysis suggest that engaging in population-based PA intervention approaches recommending the use of step counters to individuals is associated with an increase of approximately 2,000 steps per day [11]. The increase in step count is most pronounced among individuals participating in interventions aimed at promoting 10,000 steps per day [11]. Further, short-term pedometer walking interventions in primary care were associated with health benefits, such as fewer new cardiovascular events and fractures 4 years later [12]. In addition, a review and meta-analysis by Wahlich et al [13], including 9 studies in the review and 5 in the meta-analyses with follow-ups ranging from 12 months to 4 years (age range 18-89 years), revealed increases in steps per day at 12 months for intervention group participants compared with control group participants (mean difference 554, 95% CI 384-724 steps). There is also evidence of sustained intervention effects beyond 1 year. For the 2 outcomes, steps per day and minutes spent with moderate to vigorous PA (MVPA), maintained intervention effects for up to 4 years have been previously demonstrated [13].

Furthermore, previous research suggests that combining individual-level step-count monitoring interventions with environmental approaches to promote PA at the community level, such as signage in parks and other green spaces and media campaigns, in complex intervention approaches leads to increased PA at the population level [14,15]. De Cocker et al [14] implemented a multistrategy community-based intervention aimed at promoting PA in adults aged ≥18 years, which was an adaptation of the “10,000 steps Rockhampton” (Queensland, Australia) program [16,17]. This complex intervention consisted of a website to track steps, a local media campaign, environmental modifications, and the sale and loan of pedometers. Intervention effects were examined in a controlled intervention trial comparing PA at baseline and after 1 year in 872 randomly selected participants (aged 25-75 years) from an entire city (Ghent, Belgium) to 810 living in a comparison city (Aalst, Belgium) [14]. In this study, an increase of 8% in the number of individuals reaching the recommended 10,000 steps was observed in the intervention city at the 1-year follow-up compared with no increase in the comparison city. The average number of daily steps walked increased by 896 (n=660; 95% CI 599-1192) in the intervention compared with no increase in the comparison city (n=634; mean change −135; 95% CI −432 to 162). Self-reported PA confirmed this result.

A 4-year follow-up assessment of self-reported PA in 866 participants in the original trial revealed that daily step counts increased slightly in the intervention city and decreased in the comparison city. Interestingly, subgroup analyses yielded a positive interaction effect for healthier individuals and those with higher levels of education and a negative interaction effect for individuals with poor to moderate health. Thus, long-term intervention effects could not be detected at the 4-year follow-up in individuals living in the intervention city, but decreases in PA, which had been observed in the control group at the 1-year follow-up, could be prevented, except for the subgroup with poor to moderate health [15]. An evaluation of the statewide rollout of the PA intervention in Flanders compared a random sample of adults aged 25 to 75 years (n=881) with a historical control group including the baseline data of participants of both intervention and control groups of the original “10.000 Steps Ghent” intervention (n=1675). Dubuy et al [18] showed that the Flemish sample reported more walking, moderate and vigorous PA, as well as more work-related, leisure time, and household PA compared with the historical control group. In addition, the pedometer-based daily step count was higher, and a greater proportion of Flemish individuals reached the goal of 10,000 steps per day, leading the authors of the study to the conclusion that statewide socioecological complex intervention approaches can impact PA in a large population [18].

Rütten and Pfeifer [9] formulated a need for similar local and regional population-based approaches for PA promotion in Germany, pointing out that complex interventions for PA promotion, including mass media campaigns, motivational decision aids, community-based multicomponent interventions, and environmental approaches, can effectively increase PA in the general population. Thus far, 5 large German research networks (Physical Activity and Health Equity: Primary Prevention for Healthy Ageing [AEQUIPA], CAPITAL4HEALTH, Health Literacy in Childhood and Adolescence [HLCA], PartKommPlus, and SMARTACT) have been working toward improving evidence-based health promotion and primary prevention in Germany, some of them with a special focus on population-based approaches for the promotion of PA [19]. For example, the Physical Activity and Health Equity: Primary Prevention for Healthy Ageing (AEQUIPA) research network [20] conducted theory-based and participatory empirical research on different aspects of PA and healthy aging between the years 2014 and 2022 [20]. In the PROMOTE subproject of the network, multicomponent web- and print-based interventions for the promotion of PA in adults aged ≥60 years were designed following a participatory approach. They were subsequently evaluated with varying follow-ups [21-25], and intervention content and materials are publicly available for adaptation and use in future interventions [26,27].

However, additional studies are still required to strengthen the evidence base regarding effective multilevel approaches for PA promotion in a broad segment of the German population spanning a wide age range. In addition, the implementation processes involved need to be better understood. Replicating the effects of complex interventions for PA promotion, previously shown to be effective in other European countries, may help identify effective intervention strategies for the German context. Therefore, the 2 aims of this study are to adapt the previously shown to be effective complex PA intervention “10,000 Steps Ghent” to the German context (name of the German intervention: “10,000 Steps Duesseldorf”) and to evaluate its effectiveness for PA promotion in adults aged 25 to 75 years over the course of 1 year in 2 German cities in a controlled intervention study. The impact evaluation of the intervention is accompanied by a process evaluation monitoring implementation process as well as an economic evaluation, including cost-effectiveness and cost-utility analyses of the intervention. The specific research questions posed are as follows:

1. Is the intervention “10,000 Steps Ghent” adaptable and transferrable to the context of city districts in the state of North Rhine-Westphalia?
2. Is the intervention “10,000 Steps Duesseldorf” effective for the promotion of PA among residents of the districts involved?
  Do individuals located in the city that the intervention is implemented in (Duesseldorf) engage in more PA compared with individuals located in the control city (Wuppertal) after 1 year? Does the absolute number of steps taken per week increase more for individuals residing in the intervention city than in the control city?
  Does the proportion of those achieving the PA goal of 10,000 steps increase more in the intervention than in the control city after 1 year of potentially being able to participate in intervention activities?
  To what extent can these differences be attributed to the adoption, implementation, and maintenance of the intervention in the intervention city compared with the control city?
3. What are the costs associated with the provision of the intervention? Does the intervention affect health care use and indirect costs? Is the intervention cost-effective in terms of additional costs per additional person who achieves the PA goal of 10,000 steps and additional costs per additional quality-adjusted life year (QALY) gained compared between the intervention and control cities after 1 year of potentially being able to participate in intervention activities?

## Methods

### Selection of Intervention and Control City Districts, Participants, and Procedures

**Figure 1:**
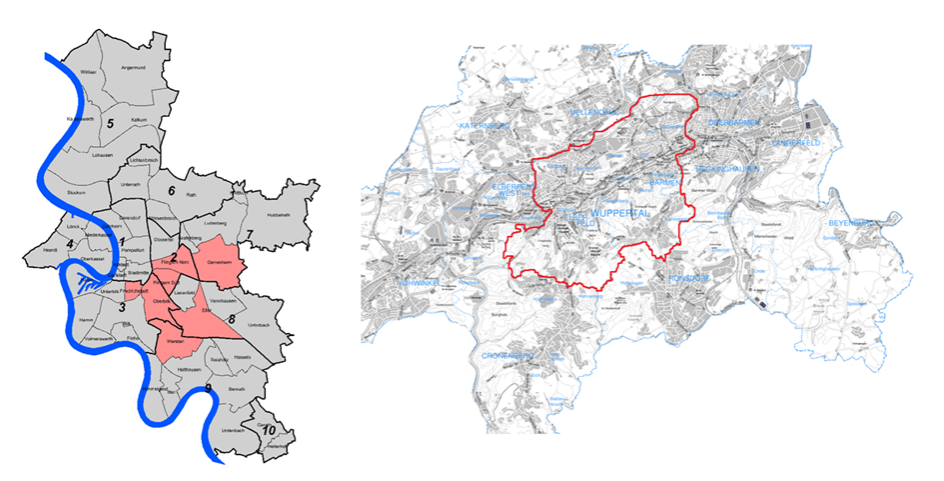
Intervention and control city district areas (left, source: Jugendamt Landeshauptstadt Düsseldorf [Youth Welfare Office, State capital Duesseldorf]; right, source: Offene Daten Wuppertal [30]).

The effectiveness of the community-based intervention for the promotion of PA is examined over the course of 1 year in a controlled intervention study. The PA behavior of individuals aged 25 to 75 years residing in the intervention districts of the city of Duesseldorf will be compared with that of individuals residing in the respective control districts of the city of Wuppertal at baseline and 1 year. Random samples were drawn based on data from the respective residents’ registration offices. As the size of the cities in North Rhine-Westphalia did not permit an assessment of all residents living in both cities, as was done in the original study [14], adjacent city districts were selected that encompass a similar number of residents as the cities included in the original study. Publicly available information on the levels of socio-spatial deprivation of the districts of Duesseldorf and Wuppertal was used [28,29] during the selection process to ensure a composition of districts balanced by the proportions of residents living in them. The socio-spatial deprivation index is composed of indicators, such as welfare benefits, living space per person, and migrant population. In Duesseldorf, the index ranges from 1 (no deprivation) to 5 (high deprivation), and in Wuppertal, the index ranges from 1 (no deprivation) to 4 (high deprivation). Corresponding to the heterogeneous population of the original study, the Duesseldorf districts of Flingern Nord, Flingern Sued, Oberbilk, Friedrichstadt, Gerresheim, Eller, and Wersten and the Wuppertal districts of Elberfeld and Barmen were chosen. The selected areas and districts in the 2 cities are shown in Figure 1 [30].

### Sampling

On March 1, 2021, a random sample of 2500 residents (25-75 years) based on postal codes (500 individuals per age group: 25-35, 36-45, 46-55, 56-65, and 66-75 years) was drawn for the Duesseldorf districts of Flingern Nord, Flingern Sued, Oberbilk, Friedrichstadt, Gerresheim, Eller, and Wersten, and 2500 residents, using the same distribution, were sampled for the Wuppertal districts of Elberfeld and Barmen. Equal proportions of men and women were included. Thus, the overarching setting in this study is the respective city district in both cities, and the subordinate settings for the implementation of the complex intervention are the organizations located in these districts (eg, senior citizen associations, sports clubs, and companies), as well as the public spaces located in them (eg, parks and green spaces).

### Recruitment

Recruitment for the study was conducted from mid-May to the end of December 2021. Individuals were informed about the study and invited to participate in it via mail. The study information, including information on data protection and the consent form (Multimedia Appendix 1), was provided in the mailed letters. Individuals interested in participating in the study were asked to return the signed consent form (and keep one for their records) and a form, including their telephone number, to the study team in the mail. Alternatively, they could contact the study team directly to make an appointment for the baseline interview and return the signed consent form after the completion of the telephone interview. However, in that case, verbal consent was provided by participants at the beginning of the telephone-based interview. In total, 3 invitation letters were sent to potential participants, and 2 reminder letters were sent 3 and 6 weeks after the first invitation letter. Once contact information was provided by a potential participant, up to 4 phone calls were made to reach the person and schedule an appointment for the baseline interview.

Owing to the COVID-19 pandemic, we expected the response rate to be extraordinarily low. To ensure that we reached the required sample sizes in both cities, a second random sample of another 4000 residents (2,000 in each city) was randomly drawn in both cities and with the same age and gender composition on July 29, 2021. During the second wave of recruitment, only 1 invitation and 1 reminder letter 4 weeks later were sent. Individuals were included in the study if they were (1) between the ages of 25 and 75 years, (2) able to understand German, and (3) residing in Duesseldorf or Wuppertal.

The baseline assessments started at the end of May 2021 and were completed at the end of March 2022. Baseline assessment entails a questionnaire completed by participants over the phone. Two study nurses and 4 student assistants underwent structured training to conduct the 1-hour telephone interviews. Data entry is done simultaneously to the interview over the secured web application, REDCap (Research Electronic Data Capture; Vanderbilt University). The respective REDCap study database is hosted at the Institute for Biometrics and Epidemiology at the German Diabetes Center Duesseldorf and is part of the regular Institute for Biometrics and Epidemiology at the German Diabetes Center IT environment with respect to data security and backups. After the interview, study participants are asked to track their PA over 7 consecutive days using the YAMAX EX210 pedometer, which records daily number of steps. In addition, they are asked to complete a wear-time diary to document the steps walked every day, the times the pedometer was worn, and the possible reasons for not wearing it. After 7 days, participants return the pedometer to the study team via mail, using a stamped return envelope provided by the study team. To validate the pedometer data, a subsample of approximately 30% of study participants receives an accelerometer (ActiGraph wCT3X-BT; ActiGraph LLC) in addition to the pedometer, which they are asked to return in a stamped return envelope after 7 days of wear time. The intervention is being implemented in Duesseldorf for 1 year since April 2022, and follow-up assessments will be conducted, starting in May 2023. The same participants who participated in the baseline assessments will be contacted again via mail, invited to participate in the telephone-based interviews again, and asked to wear pedometers (and accelerometers) for 1 week afterward. To ensure confidentiality, all person-related data assessed in this study will be pseudonymized; that is, all study participants will be assigned an ID number. The roster with the names and ID numbers will be saved in a password-protected file on the server of the Institute of Medical Sociology. The data will be stored for 10 years after the completion of the trial and deleted afterward.

### Ethics Approval

Ethics approval to conduct the study was obtained from the Ethics Committee of the Medical Faculty of the Heinrich Heine University Duesseldorf (reference number: 2021-1364; April 6, 2021). The study was registered in the German Clinical Trials Register on April 21, 2021 (trial number: DRKS00024873). All study participants are fully informed about the study and are requested to provide informed consent.

### Sample Size Calculation

**Figure 2:**
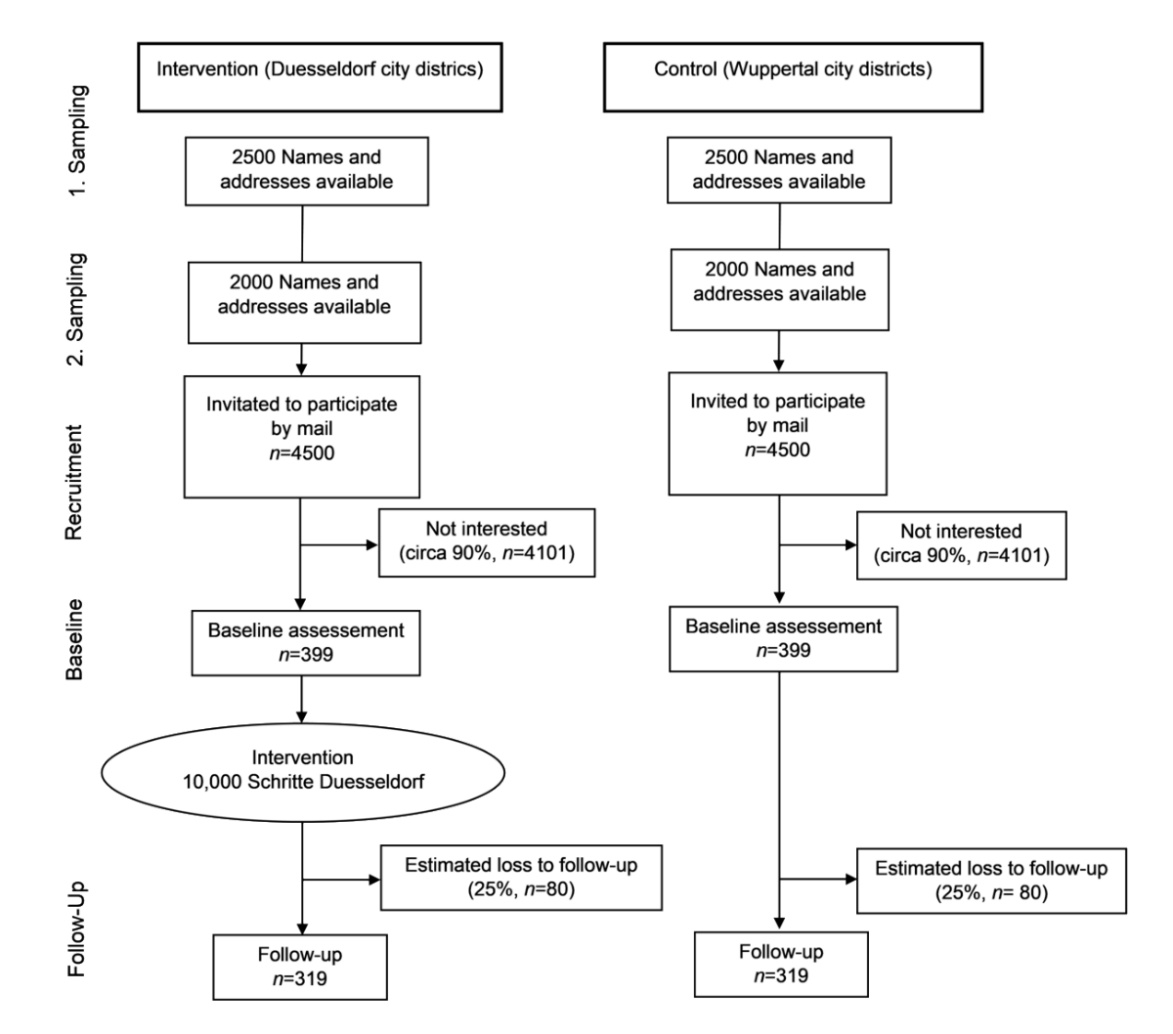
Study design.

A number of 1000 steps were assumed as the minimal relevant effect. According to the previous study by De Cocker et al [14], the SD of the number of steps is ≤4500. This ensures a power of 80% for a 2-sided *t* test at the 5% level with a total of 638 patients (SAS, version 9.4, PROC POWER, TWOSAMPLEMEANS statement). A total of 798 participants were included in the study to compensate for an expected dropout rate of 25%. It should be considered that the power calculated here represents a lower limit because a noticeable gain in power is expected in the planned analysis owing to the adjustment for baseline characteristics of participants. Figure 2 shows the study design.

### Statistical Analyses

We will use the propensity score method for statistical analysis to allow for a nonrandomized design of the study. Specifically, the primary outcome will be estimated in a weighted 2-group comparison (intervention: yes vs no). For weighting, we will use matching weights [31] that are derived from a propensity score model for the intervention effect and an exhaustive list of covariates (Multimedia Appendix 2). The analysis will be blinded for the intervention effect and performed according to the intention-to-treat principle; missing values will be replaced via multiple imputation.

### Measures and Outcomes

Sociodemographic characteristics of study participants, such as age, migration background, marital status, and socioeconomic status (including education, employment status, occupation, and income), as well as history of chronic disease and current diagnoses are assessed during the telephone interview.

### Primary Outcome

The primary outcome is the number of steps assessed via pedometers. Participants are asked to track their steps using the YAMAX EX210 pedometer, which records the daily number of steps. They are also requested to complete a wear-time diary to document the steps walked every day, the times the pedometer is worn, and the possible reasons for not wearing it. PA is also assessed using the Global Physical Activity Questionnaire in the telephone-administered questionnaire [32]. It is one of the most frequently used questionnaires for measuring PA with high levels of reliability and validity reported when comparing it with other PA questionnaires (International Physical Activity Questionnaire—Short Form and European Health Interview Survey—Physical Activity Questionnaire) [33]. On the basis of the Global Physical Activity Questionnaire, the number of minutes spent on MVPA and different types of movement will be determined (eg, for transport, see Table 1). This questionnaire was chosen because it is also used in the National Cohort (NAKO) health study [34], a large German national cohort, allowing for a comparison of PA levels of our sample to a large population-based sample.

Furthermore, a subsample of our study participants also receives an accelerometer (wCT3X-BT) to track habitual PA. Participants are instructed to position the device at their nondominant wrist during the day to capture triaxial acceleration at a sample rate of 30 Hz. Data will be processed with ActiLife (version 6.13.4) software (ActiGraph LLC), using the algorithm by Choi et al [38] to derive valid wear times with at least 4 days of a minimum 8 hours (480 minutes) wear time. Within 10-second epochs, data are analyzed using cut-off points for MVPA, energy expenditure, and metabolic equivalent of tasks, according to Freedson et al [39].

**Table 1:** Outcomes assessed in the telephone-administered interviews (part 1).

Instrument or scale and outcome				Time of assessment
**Socioeconomic characteristics**				
	**NAKO^a^ core interview [34]**			
		Date of birth	T_0_^b^	
		Country of birth	T_0_	
		Country of birth outside of Germany	T_0_	
		Time since living in the territory of the Federal Republic of Germany or DDR	T_0_	
		Nationality (German vs other)	T_0_	
		Country of birth (father and mother)	T_0_	
		Marital status	T_0_	
		In a relationship	T_0_	
		Living together with partner	T_0_	
		Living district	T_0_	
		Type of residence	T_0_	
		Number of individuals living in the household	T_0_	
		Number of individuals in the household >14 years	T_0_	
		Highest education degree obtained	T_0_	
		Types of educational qualifications	T_0_	
		Other types of educational qualifications	T_0_	
		Employment status (eg, full time or part time or unemployed)	T_0_	
		Working hours per week	T_0_	
		Previous employment status (full time or part time)	T_0_	
		Occupation	T_0_	
		Income level	T_0_	
		Average net monthly income	T_0_	
	**NAKO “Corona-questionnaire” CO-1 question no. 19 [35]**			
		Changes at work due to the COVID-19 pandemic	T_0_, T_1_^c^	
	**Self-generated item**			
		City that participant is working in	T_0_	
	**ALPHA^d^ long version** **[36]**			
		Distance to work (km)	T_0_	
	**Züll [37]**			
		Type of occupational group	T_0_	
		Self-employed or employed	T_0_	
**Built environment and PA^e^**				
	**ALPHA environmental questionnaire (long version) [36]**			
		Type of resident buildings in the immediate neighborhood	T_0_	
		Pedestrian accessibility of stores in the immediate neighborhood	T_0_	
		Infrastructure of footpaths and cycle tracks in the immediate neighborhood	T_0_	
		Quality of the living environment in the immediate neighborhood	T_0_	
		Safety in the living environment in the immediate neighborhood	T_0_	
		Attractiveness of the living environment in the immediate neighborhood	T_0_	
		Connectivity of the living environment in the immediate neighborhood	T_0_	
		Private equipment to support PA	T_0_	
		Equipment in the work environment	T_0_	
**PA**				
	**GPAQ^f^** **[32]**			
		Intensive PA during paid and unpaid work	T_0_, T_1_	
		Days of intensive PA in a usual week at work	T_0_, T_1_	
		Time of intensive PA on such a day at work	T_0_, T_1_	
		Moderate PA at work	T_0_, T_1_	
		Days of moderate PA in a usual week at work	T_0_, T_1_	
		Time of moderate PA on such a day at work	T_0_, T_1_	
		Locomotion by foot or bicycle	T_0_, T_1_	
		Days in a usual week getting around from one place to another by foot or bicycle	T_0_, T_1_	
		Time invests to get from one place to another by foot or bike	T_0_, T_1_	
		Intensive PA during leisure time	T_0_, T_1_	
		Days of intensive PA in a usual week during leisure time	T_0_, T_1_	
		Time of intensive PA in a usual week during leisure time	T_0_, T_1_	
		Moderate PA during leisure time	T_0_, T_1_	
		Days of moderate PA in a usual week during leisure time	T_0_, T_1_	
		Time of moderate PA in a usual week during leisure time	T_0_, T_1_	
		Time of sedentary behavior on a usual day	T_0_, T_1_	
		Changes of PA in the past year in general	T_0_, T_1_	
	**NAKO “Corona-Questionnaire” CO-1 question number 31 [35]**			
		Changes of PA caused by the COVID-19 pandemic	T_0_, T_1_	

^a^NAKO: National Cohort Health Study.

^b^Baseline assessment.

^c^Follow-up assessment.

^d^ALPHA: Instruments for Assessing Levels of Physical Activity and Fitness

^e^PA: physical activity.

^f^GPAQ: Global Physical Activity Questionnaire

### Secondary Outcomes

Secondary outcomes assessed at baseline and follow-up in the telephone-administered interview are described in detail in Table 2.

Briefly, at the individual level, height and weight, general self-rated health, and health behavior (smoking status, fruits and vegetables consumption, and alcohol use) are assessed. In addition, the determinants of PA are assessed (eg, social support). At the environmental level, perceptions of characteristics of the physical environment relevant for engaging in PA are assessed. To assess knowledge and awareness of intervention messages and activities spread and implemented in the intervention city districts, self-generated items are included in the follow-up questionnaire. Study participants are also asked about health care use (physician’s visits, contacts with therapists, hospital stays, and rehabilitation) retrospectively for the previous 6 months and costs pertaining to devices bought to support engagement in PA or membership fees (eg, gym membership), as well as sick leave days.

**Table 2:** Outcomes assessed in the telephone-administered interviews (part 2).

Instrument or scale and outcome				Time of assessment
**Determinants of PA^a^**				
	**GEDA^b^ [40], question no. 104 and 105 + 1 self-generated item**			
		Reasons to be or not to be physically active (including pandemic)	T_0_^c^, T_1_^d^	
	**GEDA [40], question no. 102**			
		Intention to increase PA in future	T_0_, T_1_	
	**Sudeck et al** **[41]**			
		Movement-related self-efficacy	T_0_, T_1_	
	**Self-generated items**			
		General knowledge of health benefits of PA, tracking of PA to date (use of devices; eg, pedometer, wearable, or smart watch)	T_0_, T_1_	
	**Intention to be physically active [21]**			
		How would you like to be physically active more often in the future?	T_0_, T_1_	
	**Modified version of questionnaire of Chernyak et al [42]**			
		Purchases of equipment to promote your fitness, health and well-being (past 6 months)	T_0_, T_1_	
**Health status**				
	**SF-1^e^ [43]**			
		How would you describe your health in general?	T_0_, T_1_	
	**EQ-5D-5L [44]**			
		Perceived health (eg, mobility, pain or physical discomfort, or anxiety)	T_0_, T_1_	
	**GEDA [45]**			
		Do you have a chronic illness or long-term health problem? (>6 months)	T_0_	
		Present diseases or conditions in the past 12 months	T_0_	
		Have you had high blood pressure or hypertension in the last 12 months?	T_0_	
		Have you ever been diagnosed with high blood pressure by a physician?	T_0_	
		Are you currently taking antihypertensive medications?	T_0_	
		Have you had elevated blood lipids or elevated cholesterol in the past 12 months?	T_0_	
		Have you ever been diagnosed with elevated blood lipids or elevated cholesterol by a physician?	T_0_	
		Are you currently taking medication for elevated cholesterol?	T_0_	
		Smoking habits	T_0_, T_1_	
	**GEDA [45], modified**			
		How often do you eat fruits, vegetables and salad?	T_0_, T_1_	
		How many servings of fruits, vegetables and salad do you eat per day?	T_0_, T_1_	
	**SOEP^f^-Core–2018, question no. 146 [46]**			
		What is your height in cm?	T_0_, T_1_	
	**SOEP-Core–2018, question no. 147 [46]**			
		How many kilograms do you currently weight?	T_0_, T_1_	
	**Alcohol Use Disorders Identification Test Short Version, translation from PROMOTE I [27,47]**			
		How often do you drink alcohol?	T_0_, T_1_	
		If you drink alcohol in a day, how many alcoholic beverages do you typically drink?	T_0_, T_1_	
	**Modified version of the questionnaire of Chernyak et al [42]**			
		Have you seen a physician in the last 6 months?	T_0_, T_1_	
		Which physicians have you seen in the last 6 months? (eg, general practitioner, gastroenterologist, diabetologist)	T_0_, T_1_	
		How many contacts you had in each of the last 6 months and how much time you spent in total on your outpatient?	T_0_, T_1_	
		Have you been to a therapist (eg, psychotherapist, physical therapist, speech therapist) for treatment in the last 6 months?	T_0_, T_1_	
		Which therapists have you seen in the last 6 months?	T_0_, T_1_	
		How many treatment sessions have you had in each of the last 6 months? And what is the total amount of time you spent on your treatment sessions?	T_0_, T_1_	
		Have you been hospitalized for inpatient treatment in the last 6 months?	T_0_, T_1_	
		Please indicate the reason and duration of stay for each hospitalization.	T_0_, T_1_	
		Have you been to rehabilitation in the last 6 months?	T_0_, T_1_	
		Indicate for each stay whether it was outpatient or inpatient rehabilitation. Please indicate the duration of your stay.	T_0_, T_1_	
		Please indicate how many days in total.	T_0_, T_1_	
		If your sick leave has been for more than 6 months, please indicate how many days you have been on sick leave.	T_0_, T_1_	
**Competences**				
	**German translation of ICECAP-A^g^ [48]**			
		Feeling safe and secure	T_0_, T_1_	
		Love, friendship, and support	T_0_, T_1_	
		Be independent	T_0_, T_1_	
		Performance and progress	T_0_, T_1_	
		Pleasure and enjoyment	T_0_, T_1_	
**Movement-related networks and social networks**				
	**Jackson et al [49]; Fuchs [50], adapted**			
		Perceived social support for PA by family members and friends	T_0_, T_1_	
**Perception and use of the intervention^h^**				
	**Self-generated items**			
		Awareness of the intervention, use of various intervention components	T_1_	

^a^PA: physical activity.

^b^GEDA: Gesundheit in Deutschland aktuell.

^c^Baseline assessment.

^d^Follow-up assessment.

^e^SF-1: Short Form-1.

^f^SOEP: Sozio-oekonomisches Panel.

^g^ICECAP-A: The ICEpop CAPability measure for adults.

^h^Only assessed in the intervention group.

### Intervention

#### Participatory Design

The start of the study was preceded by a 6-month participatory phase to recruit relevant stakeholders from the city of Duesseldorf to a stakeholder advisory board supporting the research team in the development and implementation of the intervention. Relevant stakeholders include representatives of the involved local communities, the public health service, the city of Duesseldorf’s administrative offices for sports and social services, 2 statutory health insurances, and the city’s Chamber of Industry and Commerce (*Industrie und Handelskammer*), as well as several companies, the physician’s chamber, and the city’s soccer club. These stakeholders were invited to participate in the board during the first 2 months of the participatory development phase. In months 3 to 6 of this phase, monthly meetings were held to adapt the Ghent intervention to the Duesseldorf context and develop a joint strategy for implementing the intervention. During these meetings, ideas concerning intervention activities and the steps necessary for implementation were brainstormed and continuously prioritized. The meetings will be continued during the implementation phase of the intervention.

#### Intervention Components

The complex intervention “10,000 Steps Düsseldorf” is a universal prevention approach aimed at motivating residents of the intervention districts of Duesseldorf to be more physically active in everyday life. The intervention components are described in further detail in the subsequent sections. In addition, Table 3 compares intervention content of “10,000 Steps Duesseldorf” to the preceding interventions in Ghent and Rockhampton.

**Table 3:** Socioecological intervention components and dissemination strategies of “10,000 Steps” studies (adapted based on Van Acker et al [51]).

	10,000 Steps Rockhampton (2-year project)	10,000 Steps Ghent (1-year pilot)	10,000 steps in Flanders	10,000 Steps Duesseldorf
Intrapersonal	Sale (GP^a^ and health services) and loan (libraries and video shops) of pedometers Website of “10,000 Steps Rockhampton”	Sale (local town shop, and health services) and loan (sport service) of pedometers Website of “10,000 Steps Ghent”	Sale and loan of pedometers in every municipality (local public services) Website updated from “10,000 Steps Ghent”	Website of 10,000 steps in Duesseldorf Provide recommendations for step counters and PA^b^ trackers on the website of “10,000 Steps Düsseldorf”
Interpersonal	Promotion of PA by health professionals and print media	Promotion of PA and distribution of folders through GPs, dietitians, physical therapists, and schools; posters in public places	Promotion of PA and distribution of folders and posters in public places Personalized contact with citizens (eg, personalized letter, mail, or phone)	Promotion of PA via step-count competitions with family and friends
Organizational	Community events and specific projects for GPs, for health services involvement, and for workplaces	Community events and specific projects for workplaces and for groups of older people	Community events and projects for the entire population and all domains of active living (PA for transport, at work, in the household, and during leisure time)	Community events and specific projects for workplaces (step-count competitions) and for groups of older people
Community	Local mass media campaign 10,000 Steps a Day—Every Step Counts Environmental: street signs, distribution of maps, and promotion of dog walking	Local media campaign 10,000 Steps a Day—Every Step Counts, 30 minutes MVPA^c^ guideline Environmental: street signs, walking circuits and billboards	Local mass media campaign in every municipality 10,000 Steps a Day—Every Step Counts, 30 minutes MVPA guideline Environmental: street signs and walking circuits	Local mass media campaign 10,000 Steps per Day—Every Step Counts Environmental: street signs and distribution of maps
Policy	Partnerships between local government and key members of community organizations, some with high-level experience in PA promotion Sale and loan of pedometers	Partnerships between the local city and provincial government, health insurance companies, and the local health promotion service Sale and loan of pedometers	Partnerships between the adopting organization and a minimum of 1 (other) local government service or 2 professional organizations Sale and loan of pedometers	Partnerships between local government and key members of community and professional organizations, health insurance companies, and commercial organizations targeting PA (eg, local soccer clubs)
Strategies for dissemination among potential adopters	Local: recruitment of community partners by researchers (microgrants) to form a local PA task force and GP training	Local: recruitment of community partners by researchers to form a local steering committee	Regional: website, mailing of the project manual and pilot study results, group meetings, displays at conferences, and e-articles	Local: recruitment of community partners by researchers to form a local steering committee; a website and e-mailing of invitations to participate, including a checklist for intervention implementation

^a^GP: general practitioner.

^b^PA: physical activity.

^c^MVPA: moderate to vigorous physical activity.

#### Intrapersonal Level: “10,000 Steps Duesseldorf” Website

The website was jointly developed with a marketing company and is based on the content of the website of the Flemish Institute of Healthy Living and Ghent University [52]. The content of the website is based on several behavior change techniques (eg, goal setting [behavior], discrepancy between current behavior and goal standard, self-monitoring of behavior, and social comparison [53]). It contains the WHO recommendations for PA and information on why and how to increase the personal daily step count, including health benefits. The target groups are individuals, groups (circles of friends and acquaintances), and organizations (eg, companies). In line with the main intervention message “Every Step Counts,” tips and information are provided on how to integrate more steps into everyday life. There are also recommendations for steps counters and PA trackers and a calendar indicating current events and offers regarding PA in the city of Duesseldorf. Visitors can create a personal profile to monitor steps, receive weekly and monthly overviews of steps, upload photos, convert other activities to steps (eg, bicycling or swimming), compare and exchange ideas with other intervention participants, and participate in step-count competitions. Information on the ongoing study examining the effects of the intervention is also included, along with references to previous projects in Ghent and Rockhampton.

#### Interpersonal Level: Promotion of PA in Groups

In step-count competitions with family and friends organized via the website, individuals are encouraged to monitor their steps, set goals, and motivate each other to engage in PA. The step-count competitions will take place as events in the entire city or in selected city districts. Step-count competitions will also take place in companies.

#### Organizational Level: Specific Projects for the Workplace and for Groups of Older People

Step-count competitions organized via the website will be advertised and implemented in companies and other organizations located in the intervention districts. Members of the stakeholder advisory board will help publicize the program and its website via email lists of companies, general practitioners, senior citizen clubs, sports clubs, and health insurance offices and by using their social media channels. In addition, print materials describing the aim and content of the intervention (brochures, flyers, and stickers), which were developed based on the print materials of 10,000 Stappen will be made available to organizations.

#### Community Level: Community Events, Local Mass Media Campaign, Environmental Street Signs, and Walking Circuits

A range of community events focusing on PA promotion are foreseen, such as the ascent of the Duesseldorf television tower (*Skyrun*), a family event in the local soccer stadium with representatives of the soccer team, and city rallies with different local themes coming from arts, culture, and history. In addition, the project will be linked to existing events, such as events organized by Duesseldorf’s administrative office for sports, city marathons, and events for health promotion organized by other entities. These events will be publicized via press releases and via the website’s social media channels and the members of the stakeholder advisory boards’ social media channels. The main intervention message, “Every Step Counts,” will be disseminated via various media channels and in public places. The aim of the media campaign is to encourage participation, as well as linking up with existing events in Duesseldorf. Flyers, as well as stickers and brochures, will be provided at the events.

In addition, for each intervention district, routes have been developed that support residents in reaching 10,000 steps per day. There is a range of 30- to 60-minute routes that can be easily integrated into everyday life and reflect the guiding principle of the project. In this context, the project’s own “Komoot” account (Komoot is an app to plan routes and socially network regarding outdoor activities) was created, on which suitable tours will be shared, which can also be shared on the website. The tours fulfill various criteria (eg, good lighting, security, accessibility, availability of seating and public toilets, and general attractiveness). Signage with routes, including information on the number of steps, and QR codes to the website will be posted in parks and public spaces of the intervention districts.

### Process Evaluation

The aim of the process evaluation is to document the frequency and intensity of the implemented intervention activities at every level of the complex intervention over the course of 1 year. This ensures intervention fidelity, meaning that it will be possible to assess whether the implementation of the intervention in Duesseldorf, Germany, was comparable with the implementation of the original intervention in Ghent. According to the RE-AIM framework [54], reach (the extent to which the program reaches the intended target group), effectiveness, adoption, implementation, and maintenance (of individual behavior change of the target group as well as long-term decision-making behavior of relevant stakeholders to consolidate intervention activities) will be assessed. A similar approach will be followed as developed by Van Acker et al [51].

For this study, the five components of the framework will be determined based on various data sources: (1) the commercial register of companies located in the intervention districts or the register of the Chamber of Commerce and Industry, (2) the individual-level data assessed in the telephone-administered interviews, and (3) a web-based questionnaire that heads of participating organizations (eg, companies) will be invited to participate in. The web-based questionnaire includes four thematic item blocks regarding (1) the characteristics of the respective organization (eg, company size) and knowledge of the intervention, (2) the adoption of the intervention and reasons for or against adoption, (3) the implementation of the intervention (eg, of the different intervention components, frequency and duration, and resources needed to implement the intervention or raise awareness), and (4) long-term intervention maintenance (reasons for or against it). Examples of the items in the web-based survey are shown in Textbox 1.

To estimate reach, first, the number of organizations potentially participating in the program in each intervention district will be determined based on the commercial register or by the Chamber of Commerce and Industry. Second, the number of organizations participating in the program will be assessed and surveyed using the web-based questionnaire. Furthermore, the proportion of individuals who were aware of intervention activities will be calculated, and to determine representativeness, age, gender, level of education, and occupation of those aware of the intervention will be compared with those unaware of the intervention. Effectiveness will be determined by comparing the 4 domains of active living (PA for transport, to work, in the household, and during leisure time activities) among individuals with and without awareness of the intervention. Adoption will be determined by estimating the proportion and representativeness of organizations implementing the intervention (compared with those not implementing the intervention). The implementation of the different intervention components (website, print-based materials, and initiation of partnerships) will be assessed, and an implementation score ranging from 0 to 100 will be calculated (see the study by Van Acker et al [51] for more details regarding the calculation of the score). To estimate maintenance, the proportion of organizations that voiced the intention to maintain the intervention will be determined.

**Example items of the web-based questionnaire used in the process evaluation.**

**Thematic block and examples of items**

1. Characteristics of the respective organization and knowledge of the intervention
  - In which organization do you work?
  - How many permanent employees does your organization have?
2. Adoption of the intervention and reasons for or against adoption
  - Did your organization participate in 10,000 Steps Duesseldorf?
  - What contributed most to the decision to carry out 10,000 Steps Duesseldorf?
  - What are the main reasons why 10,000 Steps Duesseldorf was not implemented?
3. Implementation of the intervention
  - How many employees worked on the implementation of 10.000 Steps Düsseldorf?
  - What was or is the total investment, excluding staff costs, for your organization to implement 10,000 Steps Duesseldorf?
  - Did you distribute the program materials (stickers, brochures, flyers) of 10,000 Steps Duesseldorf?
  - Which media channels did you use and at what cost?
4. Long-term maintenance of the intervention
  - Does your organization intend to plan further intervention activities in the future following the past or current 10,000 Steps Duesseldorf activities?
  - Why are no 10,000 steps Duesseldorf program activities planned for the future?

### Health Economic Evaluation

The health economic evaluation will include a cost-effectiveness analysis (CEA) and a cost-utility analysis from a societal perspective. An incremental cost-efficacy ratio (ICER; additional costs per additional person walking 10,000 steps/day) and an incremental cost-utility ratio (ICUR; additional cost for an additional QALY gained) will be determined. The outcome of the CEA is based on the primary outcome of the intervention and will be taken from the step counter measurement. QALYs will be calculated based on the EQ-5D-5L [44], which is a widely used standardized instrument to assess health-related quality of life. The EQ-5D-5L is evaluated using a German tariff to obtain preference weights [55]. As the intervention might influence people beyond health, we will also consider capability well-being using the ICEpop CAPability measure for adults (ICECAP-A) [48]. We will use a UK scoring tariff because a German tariff does not exist yet [56] and compare utilities based on capabilities with those based on QALYs.

The costs considered in the health economic evaluation comprise costs related to the development and provision of the intervention (eg, print-based materials, website, and time associated with the development of the intervention as opportunity costs), health care use (physician’s visits, contact with therapists, hospital stays, and rehabilitation), and costs associated with PA behavior (devices to support PA, membership fees [eg, gym membership], disability to work, and patient time). Productivity loss due to disability to work is calculated based on the human capital approach [57]. In a sensitivity analysis, patient time associated with PA, evaluated via opportunity costs, will also be examined. A discounting of costs and outcomes is not planned because of the short study duration.

The analysis will be performed according to the intention-to-treat principle. In the base case analysis, missing values will be imputed via multiple imputation. To make the random samples from the intervention and control region comparable, propensity score matching in the form of a weighted regression model will be applied after the imputation and for each imputed data set as suggested by Al-Janabi et al [58]. Covariates used for the matching will be the number of steps taken at baseline, age, gender, level of education, and household income.

Mean incremental costs and the mean incremental outcome of the CEA will be estimated by a using a Generalized Linear Model with gamma distribution and log link function. To estimate the mean incremental outcome of the CEA (an additional person who walks 10,000 steps/day), a Generalized Linear Model with binomial distribution and logit link function will be estimated. Bootstrap procedures will be used to calculate 95% significance intervals for the ICER and ICUR [59,60]. To investigate uncertainty surrounding the ICER and ICUR, cost-effectiveness and cost-utility planes will be generated [61,62].

## Results

Funding for this study was obtained in March 2020. Recruitment for this study and baseline data collection were conducted from May 2021 to March 2022 (as of March 2022, 626 participants were enrolled in the study). The intervention is being implemented in Duesseldorf for 1 year from April 2022 onward, and follow-up assessments will be conducted, starting in May 2023 (until September 2023). Data analysis will be performed in fall 2023, and the results will be published in spring 2024.

## Discussion

### Anticipated Principal Findings

To the best of our knowledge, this is the first study in Germany aimed at replicating the effects of a complex intervention for PA promotion previously shown to be effective in another European country. The study findings will contribute to the growing body of evidence in Germany concerning the role of complex community-based interventions for the promotion of PA in the general population [19-27]. We anticipate to obtain results similar to those reported in the original study, where adults exposed to a complex PA intervention for 1 year displayed a greater increase in daily step count (ie, average of daily steps walked increased by 896 in intervention vs no increase in the comparison city) and were more likely to reach the WHO recommendation for PA than controls (ie, 8% increase in the number of individuals reaching the recommended 10,000 steps) [14]. With regard to the expected results of the process evaluation, we foresee that the processes involved in the implementation of the various intervention components may be somewhat different, as the organizational structures of administrative offices and community organizations in Germany may operate differently compared with Belgian organizations. However, by using a similar approach to monitoring processes, we expect to be able to compare our results with those obtained in the Ghent study. Furthermore, similar to the health economic evaluation of the original intervention indicating that the intervention was cost-saving [63], we expect “10,000 Steps Duesseldorf” to be cost-effective. Other previous health economic evaluations of population-level, community-based interventions addressing PA also demonstrated cost-effectiveness [64]. Finally, previous research suggests that community-based interventions involving local stakeholders in particular may yield intervention effects after the primary evaluation is completed [13,15,16]. Our study was preceded by a 6-month participatory planning phase involving key stakeholders of PA promotion in the city of Duesseldorf. Therefore, it is conceivable that the effects may become visible after the completion of the 1-year follow-up in our study.

### Comparison With Prior Work

As outlined earlier, the primary focus is on the comparison with the Ghent- and Rockhampton-based studies. Furthermore, we expect that the recommendation and use of step counters will be associated with a similar increase in daily steps walked, as was previously reported in several reviews and meta-analyses synthesizing the results of studies examining the effects of population-based PA interventions [11,13]. The analysis of the accelerometer data of the subsample in our study will allow us to analyze weekly minutes spent on MVPA and compare the results to those reported by Wahlich et al [13] in their review and meta-analysis.

### Strengths and Limitations

A major strength of this study is the use of an innovative methodological approach for examining the effects of a complex community-based PA intervention in Germany and the ability to compare the results obtained in this study to those reported in 2 other countries in 2 continents. To our knowledge, this is the first study to examine the effect of a multistrategy population-level intervention on PA behaviors targeting individuals aged ≥18 years, which was developed based on a participatory approach. Another strength is the use of objective measurements in a subsample of the study, allowing for the validation of the questionnaire data collected in the telephone-based interviews. A limitation is that, unlike in the original study, 2 entire cities could not be compared in terms of PA behavior of participants living in them. However, by balancing the city districts of both cities included in our study using indicators of socio-spatial deprivation, we hope to have obtained a similar sociodemographic composition of our study participants. Another limitation is the ongoing COVID-19 pandemic, which complicated the recruitment of study participants. Adjustments in the number of potential study participants contacted and invited to participate were necessary to offset the difficulties in enrolling participants to the study. Finally, in our study, a trial-based health economic evaluation is conducted that does not include modeling of long-term effects and costs. Nevertheless, the health economic evaluation will provide detailed information to decision makers regarding short-term cost-effectiveness of the intervention.

### Future Directions

Should this study reveal positive effects on PA in the intervention compared with the control city districts, conducting a 4-year follow-up to assess the long-term effects of the intervention on PA and quality of life, similar to Cocker et al [15], may be useful to demonstrate long-term effects. Furthermore, comparing the results of the 3 studies conducted in Rockhampton, Ghent, and Duesseldorf may help devise recommendations for adaptation and implementation in other European cities. In Germany, a rollout of the intervention in other cities (possibly starting with the control city, Wuppertal) could be a next step after the completion of the primary evaluation. A plan for scale up or dissemination will be developed at the end of the project. In the original Ghent-based study, the intervention was rolled out in all of Flanders and evaluated based on the RE-AIM framework [14]. A similar approach could be taken after completion of this study (eg, in the entire state of North Rhine-Westphalia or in an additional German state). Following the original study by Dubuy et al [18], combined baseline data from Duesseldorf and Wuppertal from this study could serve as a historical control group. Finally, future research may also include health economic modeling techniques that could shed further light on the long-term effects of intervention participation on cost-effectiveness of this multistrategy approach for PA promotion at the community level.

## Appendix

Multimedia Appendix 1 Informed consent materials.

Multimedia Appendix 2 List of covariates in the propensity score model.

Multimedia Appendix 3 Review Proposals.

Multimedia Appendix 4 Answers to Reviewers.

## Abbreviations

AEQUIPA: Physical Activity and Health Equity: Primary Prevention for Healthy Ageing
CEA: cost-effectiveness analysis
HLCA: Health Literacy in Childhood and Adolescence
ICECAP-A: The ICEpop CAPability measure for adults
ICER: incremental cost-efficacy ratio
ICUR: incremental cost-utility ratio
MVPA: moderate to vigorous physical activity
NAKO health study: National Cohort health study
PA: physical activity
QALY: quality-adjusted life year
REDCap: Research Electronic Data Capture
WHO: World Health Organization

## Data Availability

After the completion of the study, the data can be shared with other researchers upon request but only in research collaborations with the researchers of this study. The intervention content and materials will be further disseminated via the organizations and members of the stakeholder advisory board. Trial results will be published in joint press releases and social media posts of the stakeholders, which can also be made available on the intervention website. Furthermore, the results will be published in scientific articles and presented to the public health community at scientific conferences.
