# Supplementary material for: Effects of the Population-Based “10,000 Steps Duesseldorf” Intervention for Promoting Physical Activity in Community-Dwelling Adults: Protocol for a Nonrandomized Controlled Trial
Source: JMIR Res Protoc. 2022 Sep 21;11(9):e39175. doi: 10.2196/39175 (PMC11186676; doi:10.2196/39175)
Supplement: Multimedia Appendix 1 [file resprot_v11i9e39175_app1.pdf]

UKD Universitätsklinikum Düsseldorf

hhu Heinrich Heine Universität Düsseldorf

Appendix 1. Informed consent materials.

Informed consent to participate in the study and for data processing

Please return a signed copy of this informed consent form and the consent for data processing to us in the stamped envelope provided, as well as the step counter to document the daily step count.

I have read the study information and the information regarding data protection provided to me and consent to participate in the study „10.000 Steps NRW“. I am aware that my participation is voluntary and that I can withdraw from the study anytime without any disadvantages arising to me.

In addition, I consent to being contacted again at a later point in time. At that point, I will be able to decide for or against a participation in a subsequent study.

Last name:	
First name:	
Date:	
Signature:	

<u>Please return this consent regarding data protection to us in the stamped envelope, as well as the step counter.</u>

Evaluation of the effects of the community-based complex intervention „10,000 Steps Ghent“ in two cities of Northrhine-Westfalia:

10.000 Steps NRW

I am aware of and I consent to the assessment, storage, and analysis of person-related data in this study. The use of the data regarding my health follows legal regulations and is based on my voluntarily provided consent to participate in the study. This means that I cannot participate without the consent provided below.

Consent regarding data protection

1) I consent to the assessment of data in this study, including data regarding my health, which will be stored either paper-based or using electronic storage devices at the university clinic Duesseldorf in a pseudonymized form. If necessary, the assessed pseudonymized data will be provided to the German Diabetes Center for the purpose of an efficacy evaluation. The establishment of a reference to an individual person by third parties will not be possible.

2) I have been informed that I can revoke my consent regarding recording, storage, and use of my data at any time. Following a revokation, my data will be deleted immediately.

3) I consent that my data will be stored for 10 years after the completion or discontinuation of the study. After that, my person-related data will be deleted, should there be no legal, statutory or contractural storage deadlines contrary to this action.

Name Study participant (printed letters)   Date   Signature
