# Supplementary material for: Effects of the Population-Based “10,000 Steps Duesseldorf” Intervention for Promoting Physical Activity in Community-Dwelling Adults: Protocol for a Nonrandomized Controlled Trial
Source: JMIR Res Protoc. 2022 Sep 21;11(9):e39175. doi: 10.2196/39175 (PMC11186676; doi:10.2196/39175)
Supplement: Multimedia Appendix 2 [file resprot_v11i9e39175_app2.pdf]

Appendix 2: List of covariates in the propensity score model.

**Personal and anthropometric and socioeconomic factors**	Age<br>Sex<br>Race (migration background)<br>BMI (height and weight)<br>Education level<br>Type of occupation<br>Change of work due to COVID<br>Income<br>Marital status, living with a steady partner
**Environmental factors**	Built environment
**Lifestyle factors**	Steps at baseline<br>Physical activity<br>Sedentary time<br>Alcohol consumption<br>Smoking status<br>Change of activity due to COVID<br>Determinants of physical activity<br>Fruit and vegetable consumption
**Health-related factors**	History of diabetes<br>History of CVD<br>History of hypertension<br>History of hyperlipidemia<br>History of myocardial infarction<br>History of stroke<br>History of arthrosis<br>History of back pain<br>History of neck pain<br>History of cancer<br>History of asthma<br>History of COPD<br>History of liver cirrhosis<br>History of urinary incontinence<br>History of chronic liver disease<br>History of depression<br>Allergies<br>Antihypertensive medication<br>Lipid-lowering medication<br>Quality of life<br>Self-rated health<br>Social support
