# Supplementary material for: Effects of the Population-Based “10,000 Steps Duesseldorf” Intervention for Promoting Physical Activity in Community-Dwelling Adults: Protocol for a Nonrandomized Controlled Trial
Source: JMIR Res Protoc. 2022 Sep 21;11(9):e39175. doi: 10.2196/39175 (PMC11186676; doi:10.2196/39175)
Supplement: Multimedia Appendix 3 [file resprot_v11i9e39175_app3.pdf]

DLR Projektträger / Bereich Gesundheit

Deutsches Zentrum für Luft- und Raumfahrt

DLR Projektträger
Heinrich-Konen-Straße 1, 53227 Bonn

Institut für Medizinische Soziologie
Centre for Health and Society
Medizinische Fakultät
Heinrich-Heine Universität Düsseldorf
Frau Prof. Dr. Claudia Pischke
Moorenstr. 5
40225 Düsseldorf

Ihr Zeichen
Ihr Schreiben
Unser Zeichen
Ihr/e Gesprächspartner/in Dr. Michael Ebert
Telefon 0228 3821-1739
Telefax 0228 3821-1257
E-Mail Michael.Ebert@dlr.de

25.07.2019

Announcement of the Federal Ministry of Education and Research for the funding of research to strengthen the evidence base and transfer in prevention research - "Quality in Health Research -" in the framework program Health Research of the Federal Government of 05.12.2018.

hre project outline "Evaluation of the impact of the community-based complex intervention "10,000 Steps Ghent" in two cities in North Rhine-Westphalia" of 28.03.2019

Dear Professor Pischke,

Thank you for your participation in the above-mentioned BMBF announcement. We have thoroughly reviewed your above-mentioned project outline with the involvement of an interdisciplinary review panel.

We are pleased to inform you that your project outline is recommended for funding. Please find attached the detailed evaluation of your outline. The evaluation contains the conditions as well as further recommendations and notes.

Please provide comments on the implementation of the following conditions and recommendations:

- In the intervention district, declarations of cooperation from companies and senior citizens' associations are also to be obtained.
- A statement is to be developed on how the requested participatory preliminary phase of six months is to be used to strengthen ratio preventive aspects.
- Geographic differences between intervention and control districts and their implications for the project should be addressed.

For budgetary reasons, the duration of the collaborative projects can probably begin no earlier than February 1, 2020.

Das Deutsche Zentrum für Luft- und Raumfahrt e. V. ist Mitglied der Helmholtz-Gemeinschaft. Vertreter des DLR sind der Vorstand und von ihm ermächtigte Personen. Auskünfte erteilt der Leiter Allgemeine Rechtsangelegenheiten, Linder Höhe, 51147 Köln (Hauptsitz des DLR).

Besucheradresse:
Heinrich-Konen-Straße 5
53227 Bonn
Telefon +49 228 3821-0
Internet DLR-PT.de
Zertifiziert nach ISO 9001

We would like to point out that all the above information represents an interim statement on the current status of our review of your application. The final review will only be possible once the complete documentation has been submitted.

Under no circumstances can you derive a legal claim to funding from this letter. In particular, if your formal application is rejected at a later date, you will not be reimbursed for any expenses or costs incurred.

Please inform your partners immediately about this interim result. We will also send you this letter in parallel by e-mail.

If you have any questions, please feel free to call us.

We wish you every success and look forward to working with you.

Kind regards

i. A. i. A.

Dr. Michael Ebert Isabella Schönfeld

Attachments

- Detailed evaluation of the project outline

Attachments

Request_ID	Coordination	Requested fundingl	
**QUALI_PRAEV_M_2-020**	**Pischke**	**718.772,00 €**	
Project titel			
Evaluation of the impact of the community-based complex intervention "10,000 Steps Ghent" in two cities in North Rhine-Westphalia.			

In the proposed project, the implementation and effectiveness of a community-based complex intervention to promote physical activity among adults will be evaluated over a period of one year in the cities of Düsseldorf (intervention group) and Wuppertal (control group) as part of a controlled intervention study. The original study was conducted in two cities in Belgium.

The project leaders are proven scientists in the field of prevention research, as evidenced by numerous publications, including in international journals. The topic is of great relevance not least because the intervention is easy to communicate and clear positive results were achieved in the initial study. Overall, the study design of the proposed project is absolutely convincing.

However, since Düsseldorf is already a "Healthy City," contamination effects cannot be completely ruled out. Furthermore, geographical differences between the two cities are not sufficiently taken into account. For example, Wuppertal is significantly hillier than Düsseldorf, which is why an increase in physical activity is likely to be easier to achieve there than in Wuppertal. All in all, the elements of relative prevention tend to be less pronounced. So far, there are also comparatively few cooperation commitments from practical partners (e.g., there is a lack of senior citizens' associations and companies).

Vote:

The application is recommended for funding with conditions

<u>Conditions</u>

- In the intervention district, declarations of cooperation from companies and senior citizens' associations are also to be obtained.
- A statement is to be developed on how the requested participatory preliminary phase of six months is to be used to strengthen ratio preventive aspects.
- Geographic differences between intervention and control districts and their implications for the project should be addressed.
