# Supplementary material for: Effects of the Population-Based “10,000 Steps Duesseldorf” Intervention for Promoting Physical Activity in Community-Dwelling Adults: Protocol for a Nonrandomized Controlled Trial
Source: JMIR Res Protoc. 2022 Sep 21;11(9):e39175. doi: 10.2196/39175 (PMC11186676; doi:10.2196/39175)
Supplement: Multimedia Appendix 4 [file resprot_v11i9e39175_app4.pdf]

UKD Universitätsklinikum Düsseldorf

HEINRICH HEINE UNIVERSITÄT DÜSSELDORF

Institut für Medizinische Soziologie

institut für medizinische soziologie

Universitätsklinikum Düsseldorf AöR • Moorenstraße 5 • 40225 Düsseldorf
Sektion Public Health • Gebäude 14.82

Sektion Public Health

Leitung Sektion Public Health
Univ.-Prof. Dr. Claudia Pischke
Telefon (0211) 81-08599
Claudia.Ruth.Pischke@med.uni-duesseldorf.de

Stellvertretende Leiterin des Instituts für Medizinische Soziologie
Univ.-Prof. Claudia Pischke

Sekretariat:
Szilvia Jancso-Papoutsis
Telefon (0211) 81-06418
public-health@uni-duesseldorf.de

Website des Instituts:
www.uniklinik-duesseldorf.de/med-soziologie

Website des Studiengangs Public Health
www.public-health.hhu.de

chs centre for health & society

Besucher:
Universitätsklinikum Düsseldorf
Geb. 14.82 Ebene 01
40225 Düsseldorf

Straßenbahnlinien U71, U73, U83, 704
Buslinien 731, 735, 827, 835, 836, NE7, SB56

Ansprechpartner:	Durchwahl:	E-Mail:	Datum:
Prof. Dr. Claudia Pischke	0211 81-08599	claudia.pischke@hhu.de	30.09.2019

Statement on the implementation of the conditions and recommendations for the project outline

Dear Dr. Ebert,

Herewith I am sending you our statement regarding the conditions and recommendations submitted in your letter of 07/25/2019 regarding the project outline entitled "**Evaluation of the impact of the community-based complex intervention "10,000 Steps Ghent" in two cities in North Rhine-Westphalia**" (dated 03/28/2019). The cooperation statements required as part of the conditions are also attached.

If you have any questions, please do not hesitate to contact me.

Yours sincerely

Claudia Pischke

Prof. Dr. Claudia Pischke

Comment on the implementation of the following conditions and recommendations:

1. in the intervention district, cooperation declarations from companies and senior citizens' associations should also be obtained.

Ad 1. A systematic overview of all companies located in the intervention city districts (Flingern Nord, Flingern Sued, Oberbilk, Friedrichstadt, Gerresheim, Eller, Wersten) as well as senior citizens' associations has been prepared. The senior citizens' associations in Duesseldorf call themselves centers plus and have various welfare associations as their sponsors. They are under the control of the Office for Social Affairs. We now have a declaration of cooperation from the head of the department for seniors, the disabled and those in need of care at the office, which ensures access to all centers plus in the city of Duesseldorf, including those located in the intervention city districts. In addition, there is now a declaration of cooperation from the head of the Sports Office in Duesseldorf and thus access to sports clubs and other sports facilities.

With regard to the companies, one to two companies per city district were initially informed about the study via email and subsequently in repeated telephone calls and invited to participate in the intervention. We now have two declarations of cooperation from small and medium-sized companies and one from a large company. We are in discussion with further companies from which we were unable to obtain a final declaration of cooperation in this relatively short period of time.

The majority of the relevant stakeholders in the various institutions are willing to participate in the participatory preliminary phase of the project. For example, a meeting will be held in November with the respective department heads of the Office of Social Affairs and the Office of Sports to discuss next steps for the project planning. In the course of the participatory preliminary phase, additional relevant stakeholders and institutions are identified in a snowball process together with the stakeholders who have been won over for the project so far and, if necessary, included in the steering committee for the development of the implementation strategy.

2 A statement is to be prepared on how the requested participatory preliminary phase of six months is to be used to strengthen relationship prevention aspects.

Ad 2: The intervention is per se a ratio preventive intervention in the context of which offers for the promotion of physical activity are made in the different contexts. In this way, aspects of both the social and the spatial environment are to be changed and a change in behavior or an increase in physical activity among urban dwellers is to be promoted. We aim to replicate the findings of the Belgian study. Therefore, although the original intervention will be translated and adapted for the German context, content should remain the same in order to implement an intervention comparable to the Belgian intervention with similar mechanisms of action.

Both behavioral and behavioral prevention components are included in the intervention. The use of the website and pedometers in the different contexts is more behavioral prevention, while changes in signage in the city or the creation of physical activity opportunities, including in the centers plus and businesses, are more behavioral prevention. As part of the participatory preliminary phase, in addition to the monthly meetings of the steering committee with relevant actors in the city, workshops will be offered in the various participating institutions of the intervention city districts to prepare the implementation of the intervention at the sites and to explore additional ratio changes in the various institutions and initiate their implementation. At this time, we cannot anticipate the outcomes of these workshops. For example, in the original project "10,000 Steps Ghent," active breaks were established in companies to increase daily step counts. In the city of Ghent, walkable distances were indicated by signage at various locations in the city (e.g., displaying steps from parking lots to central locations or walking routes in parks based on which 10,000 steps could be reached). Duesseldorf is a member of the WHO Healthy City Network and there is a great interest on the part of the actors in the city to establish ratio preventive changes for the promotion of physical activity in Duesseldorf (see

cooperation statements of the alderman of the Lord Mayor of the City of Duesseldorf, Prof. Dr. Meyer-Falcke, in the original application and cooperation statement of the Sports Office).

3. geographic differences between intervention and control districts and their implications for the project should be addressed.

Ad 3. We are aware that geographic differences between the two cities could influence the main outcome, i.e. the number of steps achieved. For example, it is conceivable that individuals in Wuppertal generally take fewer steps per day compared to individuals in Duesseldorf due to a more hilly environment. In order to control for any systematic differences due to variation in geographic region when analyzing the number of steps collected and comparing the intervention region to the control region, we will include the residential environment in two ways. First, we will co-survey the validated German-language built environment questionnaire by Bödeker et al. (2012) (adapted from Brownson et al., 2004) in the context of which, among other things, topography of the living environment is subjectively recorded (scale C: land use -access: "The streets in my living environment are hilly, which makes walking difficult.", response options: 1= "do not agree at all" to 4= "completely agree"). Second, we will contact the statistical offices of the two cities to record the topography or relief energy of the neighborhoods. The residential environment information will be incorporated into the propensity score model during the evaluation.

Sources:

Bödeker M, Bucksch J, Fuhrmann H. Bewegungsfreundlichkeit von Wohnumgebungen messen - Entwicklung und Einführung der deutschsprachigen „Neighborhood Environment Walkability Scale". *Präv Gesundheitsf* 2012, 7:220–226. DOI 10.1007/s11553-012-0344-3

Brownson RC, Chang JJ, Eyler AA et al. Measuring the environment for friendliness toward physical activity: a comparison of the reliability of 3 questionnaires. *Am J Public Health* 2004, 94:473–483. DOI:10.2105/ajph.94
